# The MKK-Dependent Phosphorylation of p38α Is Augmented by Arginine Methylation on Arg49/Arg149 during Erythroid Differentiation

**DOI:** 10.3390/ijms21103546

**Published:** 2020-05-17

**Authors:** Mei-Yin Liu, Wei-Kai Hua, Chi-Ju Chen, Wey-Jinq Lin

**Affiliations:** 1 Institute of Biopharmaceutical Sciences, National Yang-Ming University, Taipei 112, Taiwan; lsnancy51@gmail.com (M.-Y.L.); charleshua921@gmail.com (W.-K.H.); 2 Institute of Microbiology and Immunology, National Yang-Ming University, Taipei 112, Taiwan; cjchen@ym.edu.tw

**Keywords:** arginine methylation, erythroid differentiation, MKK3, phosphorylation, PRMT1, p38 MAPK

## Abstract

The activation of p38 mitogen-activated protein kinases (MAPKs) through a phosphorylation cascade is the canonical mode of regulation. Here, we report a novel activation mechanism for p38α. We show that Arg49 and Arg149 of p38α are methylated by protein arginine methyltransferase 1 (PRMT1). The non-methylation mutations of Lys49/Lys149 abolish the promotive effect of p38α on erythroid differentiation. MAPK kinase 3 (MKK3) is identified as the major p38α upstream kinase and MKK3-mediated activation of the R49/149K mutant p38α is greatly reduced. This is due to a profound reduction in the interaction of p38α and MKK3. PRMT1 can enhance both the methylation level of p38α and its interaction with MKK3. However, the phosphorylation of p38α by MKK3 is not a prerequisite for methylation. MAPK-activated protein kinase 2 (MAPKAPK2) is identified as a p38α downstream effector in the PRMT1-mediated promotion of erythroid differentiation. The interaction of MAPKAPK2 with p38α is also significantly reduced in the R49/149K mutant. Together, this study unveils a novel regulatory mechanism of p38α activation via protein arginine methylation on R49/R149 by PRMT1, which impacts partner interaction and thus promotes erythroid differentiation. This study provides a new insight into the complexity of the regulation of the versatile p38α signaling and suggests new directions in intervening p38α signaling.

## 1. Introduction 

The p38 mitogen-activated protein kinases (MAPKs) play pivotal roles in a number of cellular processes, such as inflammation, stress response, differentiation, and survival [1]. The four members (p38α, p38β, p38γ and p38δ) of the family share high sequence homology and common regulatory mechanisms. However, they also exhibit characteristic biochemical properties and unique cellular functions [2]. The dysregulation of p38α activity plays a dominant role in various pathological conditions. The in vitro and in vivo experiments demonstrate an essential role of p38α in various stages of hematopoiesis, including erythroid differentiation, megakaryocytic differentiation, and myelopoiesis and the involvement in hematological diseases such as myelodysplastic syndromes [3,4,5,6]. The concerted regulation of p38α with other signaling pathways can either promote tumorigenesis or suppress tumor progression as shown in colon and breast cancer, respectively [7,8]. p38α triggers the production of inflammatory mediators and cytokines, such as IL-1β and IL-6, upon stimulation [8,9]. The dysregulated activity is linked to a variety of inflammatory diseases, including chronic obstructive pulmonary disease (COPD), colitis, and rheumatoid arthritis [8,9]. The intervention of p38α signaling with an aim toward disease treatment has attracted considerable attention.

p38α MAPK is activated through a canonical cascade involving phosphorylation by upstream dual-specificity MAPK kinases (MKKs), particularly MKK3 and MKK6, on the sequence-specific threonine and tyrosine residues (Thr180 and Tyr182) located in the activation loop. This phosphorylation confers a conformational change that allows the binding of substrates and the accessibility of the catalytic center. Dual-specificity phosphatases responsible for de-phosphorylating Thr180/Tyr182 control the magnitude and duration of the signaling [2]. Accumulating evidence demonstrates the existence of alternative (or additional) mechanisms that regulate p38 activation. GRK2 (G-protein-coupled receptor kinase 2) phosphorylates p38α on Thr123 located at the docking groove for MKKs, which impairs the binding of MKK6 to p38α and diminishes the activation of p38 upon LPS (lipopolysaccharide) stimulation [10]. ZAP70 (zetachain-associated protein kinase 70) and TAB1 (transforming growth factor β-activated protein kinase 1 (TAK1) binding protein 1) can activate p38α in T cell receptor (TCR)-mediated signaling and myocardial injury, respectively [11,12]. ZAP70 phosphorylates p38α on Tyr 323, leading to autophosphorylation on Thr180 and the activation of the kinase [11]. TAB1 interacts with p38α to trigger a conformational change, leading to the autophosphorylation of p38 on Thr180 [12]. Acetylation of Lys53 in the ATP-binding pocket of p38α by PCAF/p300 increases its affinity for ATP and thus enhances the kinase activity during hypertrophy of cardiomyocytes [13]. Moreover, protein arginine methyltransferase 1 (PRMT1) promotes the activation of p38α during erythroid differentiation [3]. However, the detailed molecular mechanism is yet to be revealed. A full understanding of the regulatory mechanisms of p38α activity will provide alternative ways to develop strategies which differentiate p38α functions from other isozymes for the therapeutic needs.

PRMT1 is the predominant protein arginine methyltransferase regulating various cellular processes, including gene transcription, DNA repair, and signal transduction [14]. PRMT1 catalyzes the addition of mono- or di-methyl groups to arginine residues, leading to the alteration of protein/protein interaction, protein/nucleic acid interaction, enzymatic activity and other posttranslational modifications [15]. The malfunction of PRMT1 is tightly associated with many pathological conditions, including hematological malignancy [16,17]. PRMT1 plays important roles in hematopoiesis. PRMT1 is required for adult erythroid and lymphocyte differentiation, as shown by a conditional *Prmt1* knockout mouse model [18]. PRMT1 can methylate RUNX1, a transcription factor critical for hematopoiesis, resulting in the impairment of its association with co-repressor SIN3A, and thus impacts the maturation of myeloid and erythroid lineages in cell models [17]. PRMT1 promotes erythroid differentiation by enhancing the activation of p38α in response to EPO (erythropoietin) and AraC (1-beta-arabinofuranosyl) induction in hematopoietic CD34^+^ progenitor and K562 cells, respectively [3]. Ca^2+^ influx is an essential event in various stages during erythroid differentiation [19,20]. We have shown that Ca^2+^ up-regulates the activity of PRMT1 and stimulates erythroid differentiation via the novel Ca2^+^-PRMT1-p38α axis [20].

In this study, we identified Arg49 and Arg149 of p38α as PRMT1 methylation sites by in vitro methylation followed by mass spectrometric analysis (liquid chromatography-tandem mass spectrometry, LC-MS/MS). The non-methylation mutations of Lys49/Lys149 and Ala49/Ala149 abolished the promotive effect of p38α on erythroid differentiation. The activation phosphorylation of R49/149K mutant p38α was greatly reduced upon induced differentiation. The interaction of mutant p38α with the upstream MKK3 was significantly reduced. These results indicate that arginine methylation on R49/R149 enhances the interaction of p38α with MKK3 and thus promotes activation phosphorylation by MKK3. This study also indicates that phosphorylation by MKK3 is not a prerequisite for methylation by PRMT1 and PRMT1 acts directly on p38α. In addition, we identified that MAPKAPK2 (MAPK-activated protein kinase 2) was a p38α downstream effector involved in PRMT1-mediated promotion of erythroid differentiation. Interaction of MAPKAPK2 with p38α was also significantly reduced in the R49/149K mutant. Together, this study unveils a novel regulatory mechanism of p38α activation via protein arginine methylation on R49 and R149 by PRMT1, which impacts the interaction of p38α with its upstream kinase MKK3 and downstream substrate MAPKAPK2 and thus promotes erythroid differentiation.

## 2. Results

### 2.1. The Recombinant p38α Protein Is Methylated by PRMT1 on R49 and R149

**Figure 1:**
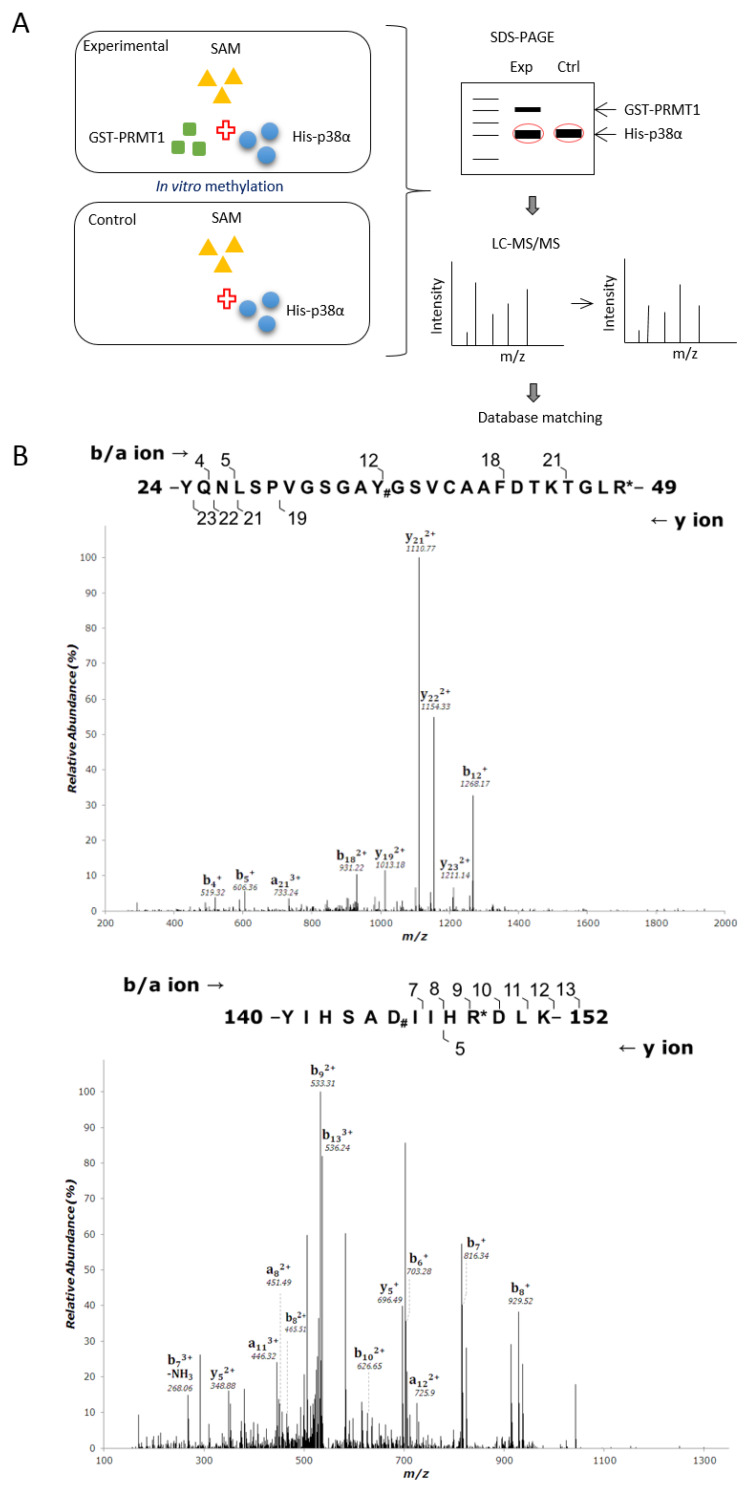

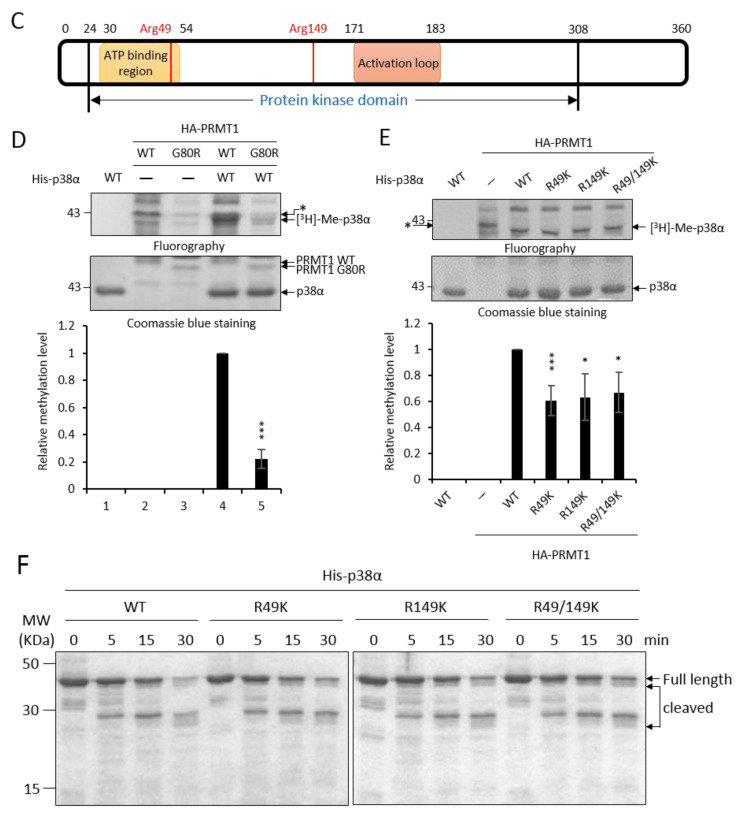
Recombinant p38α protein is methylated by PRMT1 (protein arginine methyltransferase 1) on R49 and R149. The methylation of His- p38α proteins was performed in the presence or absence of recombinant GST-PRMT1 (glutathione S-transferase-PRMT1) as described in the Methods section. Peptides containing di-methyl arginine were identified by mass spectrometric analysis as illustrated (**A**). The sequencing results of peptides harboring di-methylated R49 and R149 are shown (**B**). The localization of Arg49 and Arg149 is marked (**C**). The methylation of His-p38α was further carried out using HA-PRMT1 (hemagglutinin-PRMT1) immunoprecipitated from K562 cells. The methylation of p38α was greatly diminished with the methyltransferase-deficient mutant HA-PRMT1G80R (**D**). When R49 and R149 of p38α were mutated to K49 and K149, the methyl incorporation was significantly reduced as compared to the wild type (WT) (**E**). The quantification in (**D**) and (**E**) was carried out with results from three separate assays. Asterisks indicate non-specific bands. The recombinant p38α WT and mutant proteins (5 μg) were incubated with trypsin at 37 °C for various times and fractionated by SDS-PAGE to reveal their sensitivity to trypsin digestion (**F**). *** *p* < 0.005 and * *p* < 0.05 as compared with WT proteins.

PRMT1 promotes erythroid differentiation through enhancing the activation of p38α [3]. To investigate whether the enhanced activation of p38α is mediated by arginine methylation and the underlying mechanisms, we first performed in vitro methylation then identified methylation sites by LC-MS/MS analysis. Briefly, His- p38α was incubated with or without recombinant GST-PRMT1 (glutathione S-transferase-PRMT1) in the presence of S-adenosyl-methionine (AdoMet) as a methyl donor. The reaction mixtures were fractionated by SDS-PAGE (sodium dodecyl sulfate polyacrylamide gel electrophoresis), and gels containing p38α proteins were excised and subjected to mass spectrometric analysis. A schematic representation of the procedure is shown in Figure 1A. The sequence coverage of p38α from all identified peptides was above 70%. Peptides harboring dimethylated arginine residues on R49 and R149 in the presence of GST-PRMT1 were identified (Figure 1B) with Xcorr ≥ 2.0. These peptides were not identified when PRMT1 was absent in the methylation reactions. These results indicate that PRMT1 methylates p38α on R49 and R149. The R49 and R149 residues are located in the N lobe in the ATP-binding cleft and in the C lobe near the catalytic loop, respectively, based on the structure information of the kinase domain [21] (Figure 1C).

Methylation of p38α was further shown by using HA-PRMT1 (hemagglutinin-PRMT1) expressed in and immunoprecipitated from K562 cells. The His- p38α protein was also readily methylated by HA-PRMT1 in vitro (Figure 1D, lanes 1 and 4). The methyltransferase-deficient PRMT1G80R greatly lost the ability to methylate p38α, indicating that PRMT1 was indeed the enzyme in the immunoprecipitates responsible for the methylation of p38α (Figure 1D, lanes 4 and 5). The immunoprecipitated HA-PRMT6, which also catalyzes the formation of asymmetric di-methylarginine and shares some substrate recognition sequences with PRMT1 [14], did not methylate p38α. As reported [22], PRMT6 was readily automethylated (Supplementary Figure S1). The methyltransferase-deficient PRMT6KA mutant did not self-incorporate methyl groups (Supplementary Figure S1). These results further provide evidence for the specificity of p38α methylation by HA-PRMT1. The methylation level of p38α was significantly reduced when the arginine residues of 49 and 149 were mutated to lysine, which is not a substrate residue for PRMT1 (Figure 1E). The methyl incorporation into either R49K or R149K was reduced to around 60% compared to the wild-type p38α, indicating that both sites were methyl acceptors. The methylation on R49 and R149 may be a co-dependent event, since simultaneous mutation on R49 and R149 did not completely diminish methylation levels (Figure 1E, R49/149K). We cannot rule out the possibility that there are more methylation sites on p38α since mass spectrometric analysis does not guarantee a complete coverage of all peptides, including modified and non-modified. The structural integrity of these recombinant p38α proteins was examined by their sensitivity to protease digestion. After incubation with trypsin, all four purified proteins, WT, R49K, R149K, and R49/149K, exhibited a similar digestion pattern upon fractionation by SDS-PAGE (Figure 1F). These results indicate that R49K and R149K mutations do not cause a notable structural collapse, particularly in the surface regions.

### 2.2. The Non-Methylation R49K and R149K Mutants of p38α Lose the Ability to Stimulate Erythroid Differentiation

**Figure 2:**
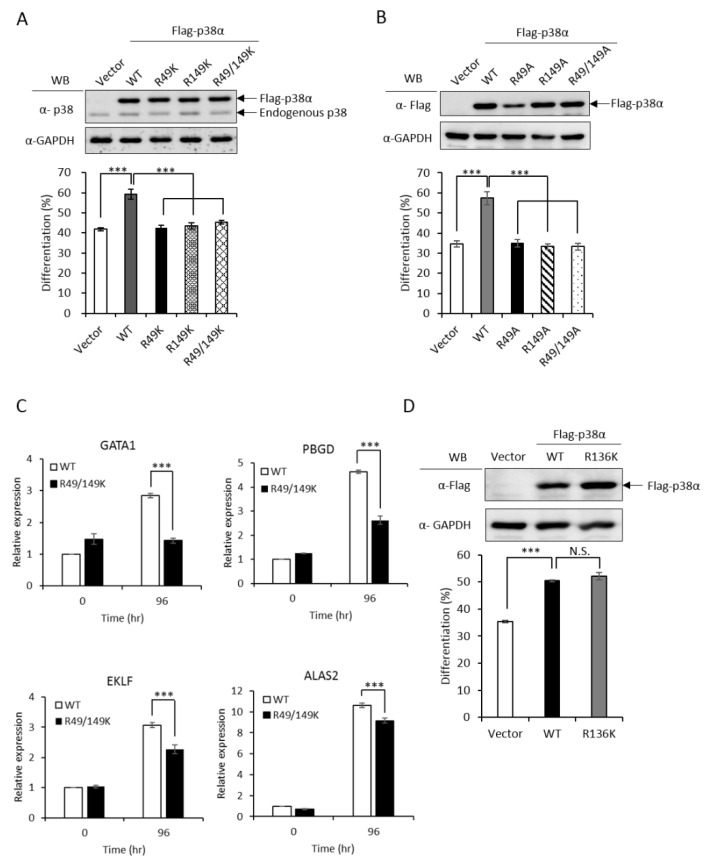
Non-methylation mutants of p38α lose the ability to stimulate erythroid differentiation. The wild-type and R49/R149 mutant p38α were expressed as Flag-tagged proteins in p38α-knockdown K562 cells. Erythroid differentiation was induced with AraC (1-beta-arabinofuranosyl) (1 μM) for 96 hours and analyzed by benzidine staining for the production of hemoglobin (**A** and **B**). Mutations of R49 and R149 to either lysine (R49K, R149K and R49/149K) or alanine (R49A, R149A and R49/149A) abolished the ability to promote differentiation (**A** and **B**). Erythroid differentiation was also analyzed by RT-qPCR for the expression of key genes. These transcripts were significantly up-regulated by AraC treatment; however, the extents were significantly reduced when R49 and R149 were mutated (**C**). The mutation of R136 to K136 (R136K) did not affect the ability to promote differentiation (**D**). All results shown are representatives of three independent experiments. Erythroid differentiation is presented as the mean ± S.E. of three repeats. *** *p* < 0.005. N.S. means no significance.

To investigate whether R49 and R149 mediate the promotive effect of PRMT1, we first examined how the non-methylation mutations (Arg to either Lys or Ala) might influence differentiation. The ectopic expression of wild-type p38α stimulated erythroid differentiation from 40% to 60% in p38α-knockdown K562 cells, as measured by hemoglobin accumulation (Figure 2A, Vector vs. WT). Both the R49K and R149K single mutants lost the ability to promote differentiation and so did the R49/149K double mutant (Figure 2A). Similarly, the stimulatory effect on differentiation was completely diminished in R49A and R149A mutants (Figure 2B). These indicate a crucial role of R49 and R149 in stimulating erythroid differentiation. Erythroid differentiation was further examined by the expression of key genes. GATA1 (GATA Binding Protein 1) and EKLF (erythroid Kruppel-like factor) are transcription factors critical for differentiation toward erythroid lineage and PBGD (porphobilinogen deaminase) and ALAS2 (5’-aminolevulinate synthase 2) are enzymes involved in heme synthesis [3]. These transcripts were significantly up-regulated by AraC treatment (Figure 2C, 0 h vs. 96 h); however, the extents were significantly reduced when R49 and R149 were mutated (Figure 2C, R49/149K). The R136 of p38α was also identified as a methylated arginine in our study. However, the methylation event was PRMT1 independent (unpublished results). The R136K mutant of p38α still retained the stimulatory effect on erythroid differentiation (Figure 2D), which provided supports for a selective role of R49 and R149 in modulating differentiation.

### 2.3. The Promotive Effect of PRMT1 on Erythroid Differentiation Is Mediated by the Methylation of p38α on R49 and R149

**Figure 3:**
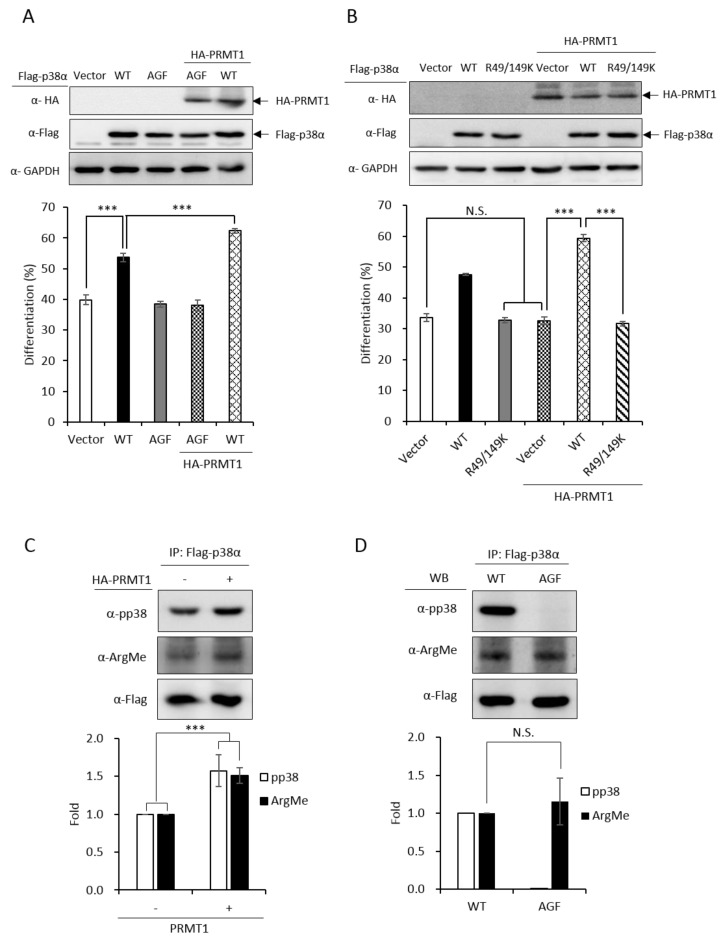
Promotive effect of PRMT1 on erythroid differentiation is mediated by methylation on R49 and R149 of p38α. The wild-type and AGF (Ala-Gly-Phe) mutant Flag-p38α were expressed in p38α-knockdown cells. The wild-type p38α (WT) promoted differentiation but the phosphorylation activation-deficient AGF mutant was unable to (**A**). HA-PRMT1 could further promote only in the presence of wild-type p38α but not the p38α AGF mutant (**A**). In the same p38α KD (knockdown) context, PRMT1 was unable to promote differentiation when R49 and R149 were mutated to K49 and K149 (**B**). Flag-p38α was expressed in the presence or absence of HA-PRMT1. After AraC stimulation, p38α was immunoprecipitated and examined by Western Blotting using anti-phospho-p38 or anti-methyl arginine antibodies. PRMT1 significantly enhanced p38α phosphorylation and arginine methylation (**C**). Upon AraC stimulation, both the WT and AGF mutant were methylated to a similar extent, although AGF was deficient in phosphorylation (**D**). All results shown are representatives of three independent experiments. Erythroid differentiation is presented as the mean ± S.E. of three repeats. The quantification in (**C**) and (**D**) was carried out with results from three separate experiments. *** *p* < 0.005. N.S. means no significance.

We have demonstrated that the PRMT1 promotes erythroid differentiation in a p38α-dependent fashion in K562 cells and human primary CD34^+^ hematopoietic progenitors [3]. To show whether the kinase activity was required, we ectopically expressed p38α in a p38α-knockdown context. Erythroid differentiation was increased from 40% to 55% (Figure 3A, Vector vs. WT). The AGF p38α mutant is deficient of kinase activity due to mutations on the Thr-Gly-Tyr motif to Ala-Gly-Phe and cannot be activated by phosphorylation via up-stream MKKs [2]. This mutant completely lost the ability to promote differentiation (Figure 3A, Vector vs. AGF), indicating a requirement for the kinase activity to promote differentiation. The catalytic activity of PRMT1 is also required to promote differentiation since the methyltransferase-deficient G80R mutant could not promote [3], indicating that PRMT1 promotes differentiation through the arginine methylation of downstream effector substrates. Notably, PRMT1 promoted differentiation only in the presence of wild-type p38α but not the p38α AGF mutant (Figure 3A, PRMT1 + WT vs. PRMT1 + AGF), indicating that the kinase activity of p38α is essential to mediate the effect of PRMT1. Together, these results provide further evidence suggesting that PRMT1 modulates the kinase activity of p38α likely via arginine methylation.

PRMT1 could not promote differentiation in a p38α-knockdown context (Figure 3B, Vector vs. PRMT1 + Vector). The co-expression of wild-type p38α greatly stimulated differentiation from around 35% to around 60% (Figure 3B, PRMT1 + Vector vs. PRMT1 + WT); however, PRMT1 was unable to stimulate when the R49/R149 of p38α were mutated to K49/K149 (Figure 3B, R49/149K vs. PRMT1 + R49/149K), indicating that the R49 and R149 of p38α mediates the stimulatory effect of PRMT1. Since the methyltransferase activity of PRMT1 is required for the promotive effect of PRMT1 [3] and PRMT1 methylated p38α on R49 and R149 (Figure 1), these results together suggest that R49 and R149 mediate the promotive effect of PRMT1 on erythroid differentiation through arginine methylation.

We further examined the phosphorylation and methylation states of p38α upon AraC stimulation. The Flag-p38α was expressed in PRMT1-knockdown cells with or without the overexpression of HA-PRMT1. Flag-p38α was immunoprecipitated from cells after AraC stimulation and examined by Western Blotting using anti-phospho-p38 or anti-methylarginine antibodies. Our results clearly show that p38α was methylated in cells and PRMT1 enhanced activation phosphorylation as well as the arginine methylation of p38α by around 1.6 folds (Figure 3C), supporting our notion that PRMT1 enhances activation of p38α by arginine methylation of the kinase. To examine whether phosphorylation was a precedent event for arginine methylation, we compared the arginine methylation status of WT and phosphorylation-deficient AGF mutant p38α. The Flag-p38α proteins were expressed in p38α-knockdown cells, immunoprecipitated after AraC stimulation and examined by Western Blotting. The WT p38α was readily phosphorylated while the AGF mutant, as expected, was not phosphorylated (Figure 3D). In spite of the dramatic difference in phosphorylation status, the arginine methylation levels were very similar in WT and AGF mutant proteins (Figure 3D), indicating that phosphorylation is not a prerequisite for arginine methylation by PRMT1.

### 2.4. The R49 and R149 Residues Play a Crucial Role in the Activation of p38α

**Figure 4:**
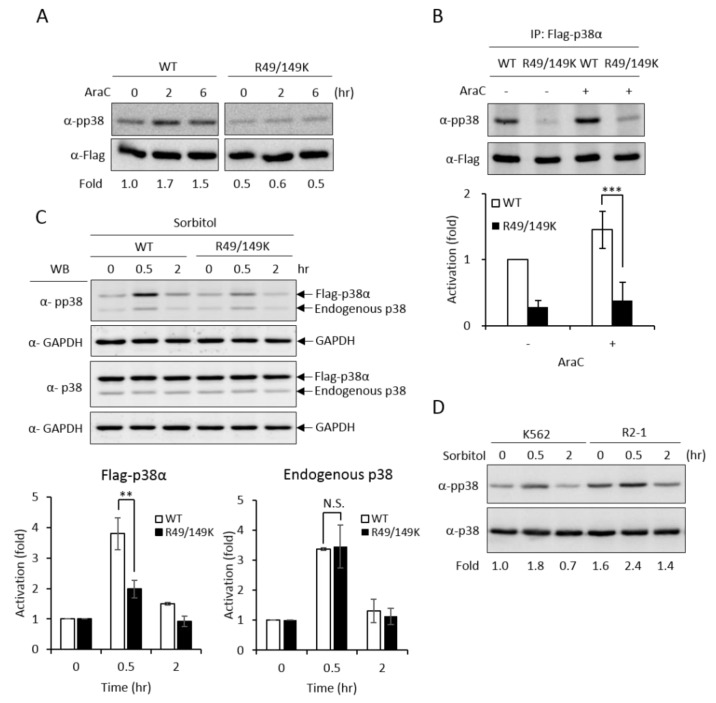
R49 and R149 residues play a crucial role in the activation of p38α. The Flag-p38α wild type or R49/149K mutant were expressed in p38α-knockdown cells. Cells were stimulated with AraC (1 μM) and the activation of p38α was examined directly by Western Blotting using specific anti-phospho-p38 antibodies (**A**) or after immunoprecipitation (**B**). Phosphorylation was greatly reduced in R49/149K. Alternatively, the cells were treated with sorbitol (150 mM) and the activation of p38α was examined by Western Blotting (**C**). The overexpression of PRMT1 (R2-1) enhanced the activation of p38α upon sorbitol stimulation (**D**). The levels of pp38 and p38 were quantified by Multi-Gauge V3.0 analysis. All results shown are representatives of three independent experiments. Statistical analysis was performed with results from three separate experiments. *** *p* < 0.005 and ** *p* < 0.01 as compared with WT.

Upon stimulation, p38 MAPK is activated by phosphorylation on the characteristic Thr-X-Tyr motif [2]. AraC treatment stimulated phosphorylation of the wild-type p38α (Figure 4A, WT); however, the activation of the R49/149K mutant was greatly reduced (Figure 4A, R49/149K), as examined by Western Blotting analysis using anti-phospho-p38 antibodies. We further immunoprecipitated Flag-p38α proteins and examined the activation phosphorylation. Similarly, R49/149K mutation significantly reduced AraC-induced activation (Figure 4B). These results indicate that R49/R149 have a role in modulating the activation of p38α. To further assess whether R49 and R149 modulate the activation of p38α in conditions other than AraC-induced differentiation, we stimulated K562 cells with sorbitol, which is a well-known strong stimulator of p38α in hyperosmotic stress [23]. The activation of p38α wild type was stimulated by around 4.0 folds at 0.5 h; however, it was reduced to around 2 folds in R49/149K mutant (Figure 4C). As an internal control, the activation of endogenous p38α was similar, around 3.5 folds, in cells expressing either Flag-p38α WT or Flag-R49/149K mutant (Figure 4C). To examine whether PRMT1 has a role in the activation of p38α induced by sorbitol treatment, we used K562 and PRMT1-overexpressing R2-1 cells as a comparison and found that the sorbitol-stimulated activation of p38 was significantly higher when PRMT1 was overexpressed (Figure 4D, K562 vs. R2-1). The basal level of p38α phosphorylation in R2-1 cells was higher than K562 parental cells before stimulation (Figure 4D, 0 h), which is in agreement with our previous observations [3]. This is conceivable because the ectopically expressed PRMT1 is active [3,4,20]. Together, these results indicate that PRMT1 likely also plays a role in enhancing the activation of p38α via methylation on R49 and R149 upon sorbitol stimulation.

### 2.5. PRMT1 Acts Downstream of MKK3 to Promote Erythroid Differentiation and the R49/149K Non-Methylation Mutant p38α Exhibits a Reduced Interaction with MKK3

**Figure 5:**
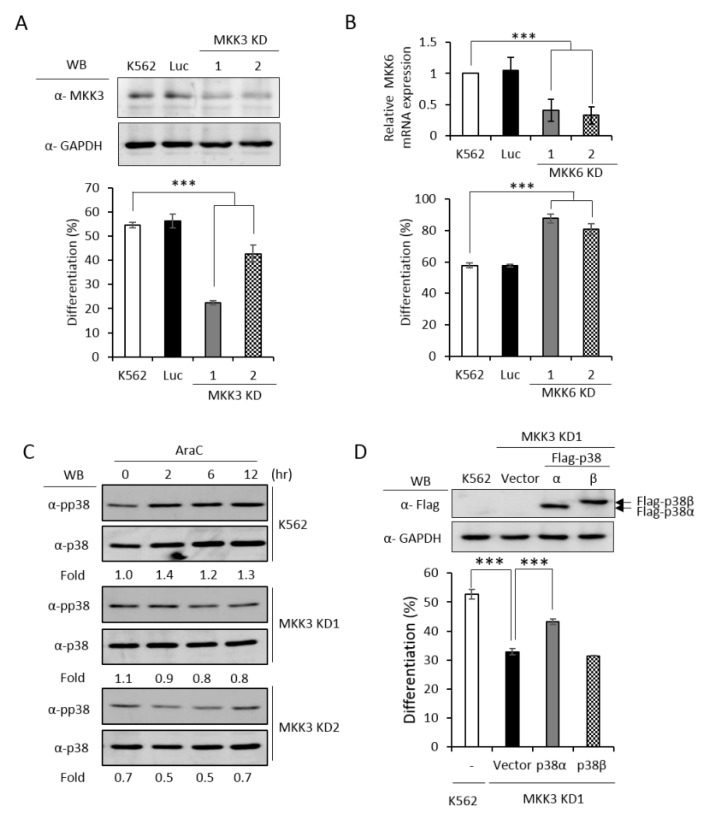

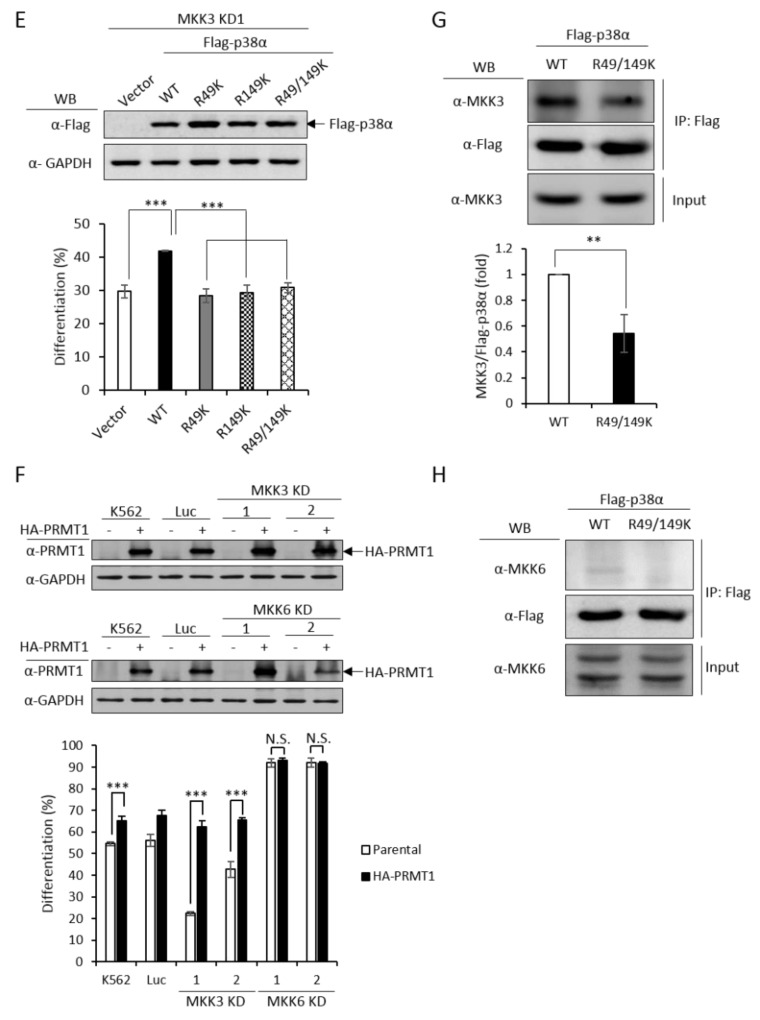
PRMT1 acts downstream of M KK3 (MAPK kinase 3) to promote erythroid differentiation, and the R49/149K non-methylation mutant exhibits a reduced interaction with MKK3. MKK3 and MKK6 were knocked down (**A**) and (**B**) as described in the Methods section. AraC-induced erythroid differentiation was significantly suppressed in MKK3-knockdown (KD1 and KD2) cells (**A**); however it was stimulated in MKK6-knockdown (KD1 and KD2) cells (**B**). The activation of p38 was significantly reduced in MKK3 KD1 and KD2 cells (**C**). Ectopic expression of p38α, but not p38β, in MKK3 KD1 cells could partially rescue differentiation (**D**). However, the R49K, R149K and R49/149K mutants lost the ability to promote differentiation (**E**). Ectopic expression of HA-PRMT1 promoted differentiation in parental K562, Luc and MKK3 KD1 and KD2 cells but had no effect in MKK6 KD1 and KD2 cells (**F**). Luc is the vector control cell. To examine the interaction of p38α with MKK3 and MKK6, Flag-p38α wild type and R49/149K mutant proteins were expressed in p38α KD cells. After cells were stimulated with AraC (1 μM) for 5 h, Flag-p38α proteins were immunoprecipitated and the protein levels of MKK3 (**G**) or MKK6 (**H**) in the immunoprecipitates were examined by Western Blot. The levels of MKK3, pp38 and p38 were quantified by Multi-Gauge V3.0 analysis. All results shown are representatives of three independent experiments. Statistical analysis was performed with results from three separate experiments. *** *p* < 0.005. ** *p* < 0.01.

MKK3 and MKK6 both are upstream kinases, which phosphorylate and activate the p38 MAPK pathway [1]. To identify which MKK is responsible for activating p38α and promoting erythroid differentiation, we established MKK3 and MKK6 knockdown cell clones (Figure 5A,B, upper) and found that only MKK3 knockdown (MKK3 KD) greatly compromised erythroid differentiation (Figure 5A, lower), indicating that MKK3 is the major MAPK kinase in AraC-induced erythroid differentiation. On the contrary, MKK6 knockdown (MKK6 KD) exhibited a remarkable stimulation on erythroid differentiation (Figure 5B, lower), indicating a previously unknown role in negatively regulating erythroid differentiation. This result suggests that MKK6 is not a p38α activating MAPK kinase in erythroid differentiation. Furthermore, the AraC-induced activation of p38 was greatly reduced in MKK3 KD cell clones (Figure 5C), confirming that MKK3 plays a role in activating p38 during differentiation. This notion was further supported by the observation that overexpression of p38α in MKK3 KD cells partially rescued the differentiation but p38β, which is not involved in erythroid differentiation [3], could not (Figure 5D). Mutations of R49 and/or R149 lost the ability to compensate the inefficiency of MKK3 activity in MKK3 KD1 cells (Figure 5E), suggesting R49 and R149 are required to mediate the activation of p38α by MKK3.

Since PRMT1 enhanced the activation of p38α (Figure 3C), we next examined whether PRMT1 acts upstream or downstream of MKK3 to enhance the activation of p38α and to promote differentiation. We overexpressed PRMT1 in MKK3 KD cells and found that erythroid differentiation was greatly stimulated to around 65% as compared to 20–40% without PRMT1 overexpression (Figure 5F). These results indicate that higher PRMT1 levels can compensate the inefficiency of MKK3 functions and suggest that PRMT1 acts downstream of MKK3. In agreement with the notion that MKK6 was not the upstream kinase for p38α (Figure 5B), PRMT1 did not further stimulate differentiation in MKK6 KD cells (Figure 5F). Taken together, these results indicate PRMT1 acts downstream of MKK3 and likely by directly methylating p38α on 49 and R149.

The MAPKs are known to form a protein complex with upstream kinases, scaffold proteins and phosphatases to bring the players to a close proximity, which confers temporal and spatial regulation of signal transduction [1]. When immunoprecipitated from cells, the p38α protein interacted with MKK3 only but not MKK6 (Figure 5G,H, WT), supporting our notions that MKK3, but not MKK6, is the major MAPK kinase for p38α. The non-methylation p38α R49/149K mutant exhibited a significantly reduced interaction with MKK3 by around 50% (Figure 5G, R49/149K). Similar to the wild type, the R49/149K mutant did not interact with MKK6 (Figure 5H, R49/149K). The expression levels of MKK3 and MKK6 were similar in the parental and R49/149K mutant cells (Figure 5G,H, input). Together with the observation that the activation of the R49/149K mutant was significantly lower than the wild type (Figure 4A–C), our results suggest that R49 and R149 up-regulates the activation of p38α through enhancing the interaction of p38α with MKK3 via arginine methylation by PRMT1.

### 2.6. Identification of MAPKAPK2 as a Downstream Effector of P38α During Erythroid Differentiation and the R49/149K Non-Methylation Mutant Exhibits a Reduced Interaction with MAPKAPK2

**Figure 6:**
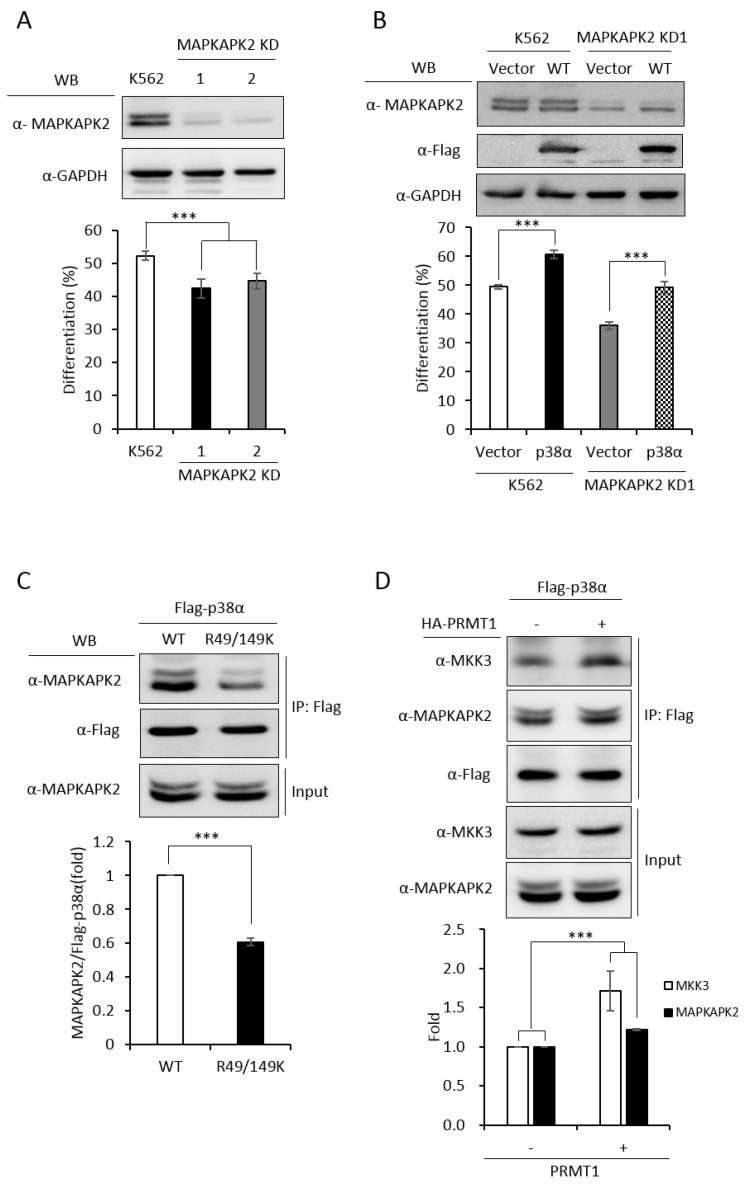
MAPKAPK2 is a downstream effector of p38α and the R49/149K non-methylation mutation reduces the interaction of p38α with MAPKAPK2. The wild-type and R49/149K mutant p38α proteins were expressed in p38α KD cells. Cells were stimulated with AraC and Flag-p38α was immunoprecipitated using anti-Flag antibodies. The interacting proteins were analyzed by mass spectrometric analysis. MAPKAPK2 was identified as an interactor of the p38α by Ingenuity Pathway Analysis (IPA). AraC-induced erythroid differentiation was reduced in MAPKAPK2 knockdown (KD) cells (**A**). The ectopic expression of p38α rescued differentiation of MAPKAPK2 KD1 cells (**B**). The Flag-p38α WT and R49/149K proteins were immunoprecipitated after AraC stimulation. MAPKAPK2 interacted with wild-type p38α; however, the R49/149K mutant exhibited a significantly lower interaction with MAPKAPK2, as examined by Western Blotting (**C**). PRMT1 promoted the interaction of wild-type p38α with MKK3 and MAPKAPK2 (**D**). The intensity of protein bands in Western Blots were quantified by Multi-Gauge V3.0 analysis. All results shown are representatives of three independent experiments. Statistical analysis was performed with results from three separate experiments. *** *p* < 0.005.

In order to have a comprehensive understanding of the biochemical and functional role of R49 and R149 methylation, we further analyzed the protein interactors of the wild-type and mutant p38α proteins. We co-expressed either the wild-type or the R49/149K mutant with HA-PRMT1 in p38α KD cells, performed immunoprecipitation, fractionated the immunoprecipitates by SDS-PAGE and carried out mass spectrometric analysis to reveal the interacting proteins. A total of 163 proteins were found to interact with both WT and mutant p38α. We then analyzed the connection of these proteins with the p38α pathway by using Ingenuity Pathway Analysis (IPA). MAPKAPK2 and MAPKAPK3 (MAPK-activated protein kinases 2 and 3) were identified by the software within the limit of “direct interaction” and “experimentally observed”. MAPKAPK2 is a known substrate of p38α [24], whereas the role of MAPKAPK3 is less described. Since, to the best of our knowledge, there was no report for the role of MAPKAPK2 in erythroid differentiation, we knocked down MAPKAPK2 and examined AraC-induced erythroid differentiation. The results showed that the knockdown of MAPKAPK2 reduced erythroid differentiation, from 50% to 35–40% (Figure 6A,B), suggesting a role of MAPKAPK2 in erythroid differentiation. The ectopic expression of p38α still promoted differentiation in the MAPKAPK-2 KD cells, likely due to the remaining low level of MAPKAPK2. However, the differentiation was significantly lower in KD-1 cells (49%) than in the K562 parental cells (61%), where the MAPKAPK2 level was normal (Figure 6B). These results suggest that p38α signals through MAPKAPK2 to promote differentiation. This notion was further supported by the immunoprecipitation experiments, showing the interaction of MAPKAPK2 with p38α (Figure 6C, WT). Notably, the interaction was significantly reduced to about 60% when R49 and R149 were mutated (Figure 6C, R49/149K). Although R49/149K mutation reduced the interaction of p38α with its upstream kinase MKK3 as well as with its downstream substrate MAPKAPK2, the interaction of p38α with protein phosphatase 2A (PP2A) was not significantly affected (Supplementary Figure S2). To examine the influence of arginine methylation on partner interaction, we immunoprecipitated Flag-p38α in the presence or absence of HA-PRMT1 upon AraC stimulation. The results show that PRMT1 enhanced the interaction of p38α with MKK3 and MAPKAPK2 by 1.7 folds and 1.2 folds, respectively (Figure 6D). Together, our results indicate that the R49 and R149 of p38α are critical for a previously unidentified role in selective partner interactions through arginine methylation by PRMT1.

**Figure 7:**
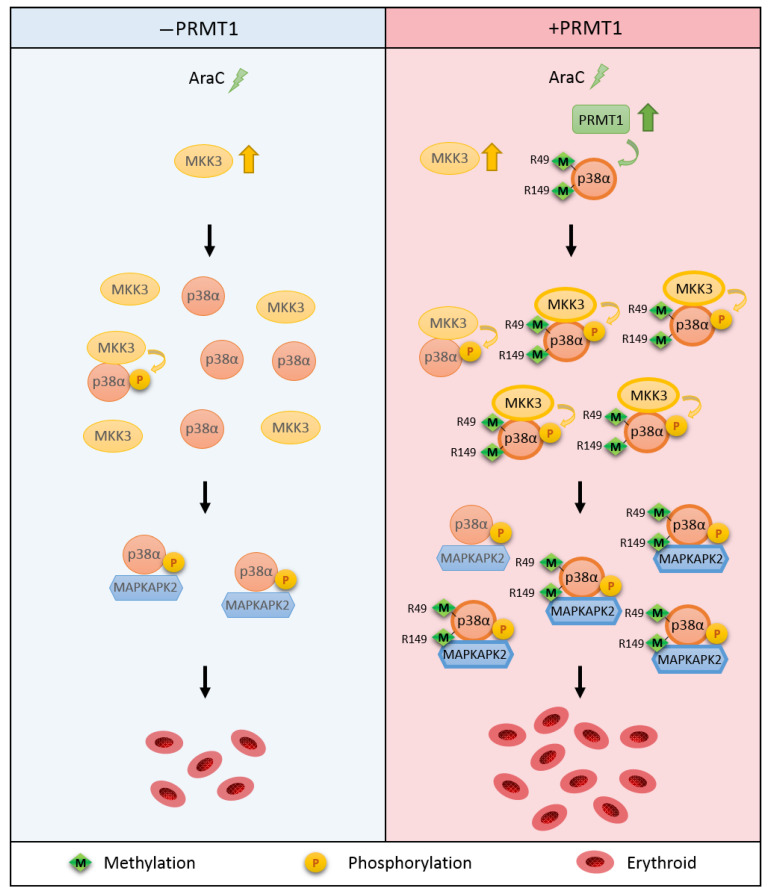
Illustration of the novel regulatory mechanism for p38α through arginine methylation on R49/R149 by PRMT1. AraC treatment stimulates the methyltransferase activity of PRMT1, which methylates p38α on R49 and R149. This facilitates the interaction of p38α and MKK3 and enhances the activation phosphorylation of p38α by MKK3. The interaction of p38α with downstream effector MAPKAPK2 is also increased upon R49/R149 methylation by PRMT1 methylation. Together, erythroid differentiation is promoted due to the facilitated p38α signaling. Green arrow: methylation. Yellow arrow: phosphorylation.

This study reveals a novel regulatory mechanism for p38α. Arginine methylation of R49/R149 by PRMT1 occurs upon stimulation, which does not require a prior phosphorylation on Thr180 and Tyr182. The methylation of R49/R149 facilitates the selective association of p38α with MKK3 and thus augments phosphorylation by MKK3 and enhances the activation of p38α. The methylation of R49/R149 also facilitates the association of p38α with a downstream effector MAPKAPK2, which enhances the propagation of signals to up-regulate erythroid differentiation. An illustrated model is presented in Figure 7.

## 3. Discussion

Extensive efforts have been focused in manipulating the activity of p38 MAPK for therapeutic purposes due to its close association with various pathological conditions, including inflammatory and malignant diseases [8]. Activation by phosphorylation via the upstream MKKs is the canonical mode of kinase activation. This study identifies, for the first time, a posttranslational modification of arginine methylation on Arg49 and Arg149 of p38α by PRMT1 and demonstrates that methylation on R49/R149 modulates activation of p38α through an increased association with the upstream MKK3 and the downstream effector MAPKAPK2 and thus impacts erythroid differentiation. This study elucidates a novel regulatory mechanism for p38α activation and indicates a potential new strategy in intervening p38α signaling.

Upon stimulation, a cascade of phosphorylation events involving MEKs and MKKs result in the phosphorylation of p38 MAPKs on the conserved Thr^180^-Gly-Tyr^182^ motif, leading to an open and extended activation loop, which allows for the binding of substrate and facilitates catalysis [21]. In addition to this canonical mode, increasing evidence has shown other modes of regulation. The phosphorylation of Thr180/Tyr182 of p38 can also be achieved by p38 auto-catalysis in response to T cell receptor (TCR)- or TNFα-mediated signaling in an MKK-independent manner [11,12]. The triggering of autophosphorylation can be induced by a precedent phosphorylation of p38 on Tyr323 by ZAP70, a T cell receptor-associated tyrosine kinase [11], or by the binding of TAB1, an adaptor protein in TNFα-mediated signaling, with p38α [12]. In the MKK-dependent mode, docking regions on p38 mediate its interactions with various partners including MKKs, substrates, and phosphatases, in a context-dependent fashion, and contributes to the selective transduction of diverse signals [25]. GRK2 (G-protein-coupled-receptor kinase 2) phosphorylates p38, in a known docking groove, compromises the binding of MKK6 and thus suppresses the activation of p38 upon LPS stimulation in macrophages [10]. In this study, we show that arginine methylation of R49 and R149 by PRMT1 enhances the activation phosphorylation of p38α upon induced erythroid differentiation (Figure 3C, Figure 4A,B). We identify that MKK3 is the responsible upstream kinase (Figure 5A,C,D and E). The enhanced activation of p38α is mediated by an increased association with the upstream kinase MKK3 via R49/R149 methylation (Figure 5F,G). MKK6 is not an upstream kinase for p38α in AraC-induced erythroid differentiation (Figure 5B,F) and is not associated with p38α no matter whether R49 and R149 are wild-type or mutated (Figure 5H). These results uncover a novel mechanism for the regulation of p38α signaling selectively through MKK3 by arginine methylation.

The correct recognition of MAPKs by the cognate interacting partners is critical for specifically transducing signals with a high fidelity. A common docking (CD) domain containing a few of conserved acidic residues is found in MAPKs that contribute mainly to the binding affinity [25,26]. The intervening sequences of these acidic residues and sequences in other regions play predominant roles in partner selectivity [25,26]. The sequence context for partner selection and binding affinity in response to different stimuli is not fully understood. Arg49 and Arg149 are not located near the conserved acidic residues Asp313, Asp315, and Asp316 in the CD domain of p38α. Arginine methylation catalyzed by PRMTs alters the hydrogen bonding capacity, hydrophobicity, and steric hindrance of the target arginine and its vicinity [27] and thus affects a wide range of protein properties. The activity of PRMT1 is up-regulated during erythroid differentiation [3], leading to the methylation of p38α and the enhanced activation of the kinase (Figure 3C). Arginine methylation on Arg49/Arg149 can potentially cause a conformational change or an increased hydrophobicity favoring partner selection and/or binding affinity. In addition, the HRD sequence (His148-Arg149-Asp150) is shown to lock p38α in an inactive conformation that can be disrupted by Tyr323 phosphorylation [28]. This observation raises the possibility that the methylation of R149 may facilitate a conformational change favorable for kinase activation. Arg49 is stereoscopically near Leu108 and Met109, which is part of a lipophilic pocket facilitating ATP binding [29]. The possibility that methylation on R49 favors the stabilization of this lipophilic pocket is worthy of further study.

p38α is a versatile MAPK participating in many important physiological and pathological conditions [1,2,3]. The molecular events involved in its activation and regulation have attracted considerable research attention. A number of posttranslational modifications (PTMs) in p38α have been identified via proteomics approaches [30]. However, relatively few of the identified PTMs are reported with functional impacts. In this study, we have identified several methylated arginine residues in p38α. Among those, R49 and R149 are dimethylated only in the presence of PRMT1, suggesting they are PRMT1 substrate sites (Figure 1). Non-methylation mutants of R49K and R149K lost around 40% of methyl incorporation (Figure 1), indicating the existence of other potential PRMT1 sites. In cells, the activation phosphorylation of R49/149K mutant is remarkably reduced (Figure 4), which explains the incapability of the mutant to promote erythroid differentiation (Figure 2). The lack of Thr^180^-Gly-Tyr^182^ phosphorylation of the AGF mutant does not affect its arginine methylation level (Figure 3D), indicating that phosphorylation is not a prerequisite for methylation. Arginine methylation by PRMT1 significantly increases the association of p38α with the upstream kinase MKK3 (Figure 5G and Figure 6D) and the downstream substrate MAPKAPK2 (Figure 6C,D). This study reveals that the arginine methylation of p38α on R49/R149 by PRMT1 renders the kinase more accessible to the enzyme (MKK3) and also facilitates its action toward the downstream effector MAPKAPK2. The importance of methylation in the MAPK signaling is also evidenced in other studies. The methylation of MAPK kinase kinase 2 (MAP3K2) on Lys260 by SMYD3 (SET and MYND domain containing 3) blocks its interaction with the negative regulator PP2A phosphatase and activate Ras-mediated MEK/ERK (extracellular signal-regulated kinase) signaling [31]. The arginine methylation of Raf by PRMT5 results in an increased degradation, a decreased Ras-Raf-Erk signaling and a reduced proliferation of PC12 cells upon EGF (epidermal growth factor) stimulation [32]. Our results demonstrate that, in addition to erythroid differentiation, the sorbitol-stimulated activation of p38α is also enhanced by PRMT1 and dependent on the methylation of R49 and R149 (Figure 4C,D). Together, methylation, in collaboration with phosphorylation, is emerging as a critical regulatory mechanism of the MAPK pathways via which extracellular cues are integrated to elicit an appropriate response in terms of signal magnitude, duration and specificity.

Although p38α has been shown to be a critical player in various stages during erythroid differentiation, its upstream activating kinase and downstream substrate had not been clearly and fully revealed. Our results decisively show that only MKK3, not MKK6, mediates the activation of p38α in AraC-induced erythroid differentiation (Figure 5A,B). The immediate downstream effectors of p38α in erythroid differentiation are much less described. We identify that MAPKAPK2 participates in erythroid differentiation via p38α signaling (Figure 6). The RNA-binding protein human antigen R (HuR) protein is an effector of MAPKAPK2 that can bind and stabilize GATA1 transcripts during embryonic erythropoiesis [33]. GATA1 is a critical transcription factor upregulated during erythroid differentiation [34] in a p38α-dependent fashion (Figure 2C). Whether MAPKAK2 promotes erythroid differentiation by stabilizing GATA1 transcripts via HuR is of interest.

Genetically modified mice have evidenced that the p38α pathway plays important roles in inflammatory responses and hematopoietic homeostasis among others [8]. A number of pharmacological inhibitors of p38 MAPK have been developed and tested in clinical trials for treating inflammatory diseases, such as rheumatoid arthritis and chronic obstructive pulmonary disease and hematopoietic diseases, such as myelodysplastic syndromes (MDS), which frequently leads to hematological malignancy [35]. Major drawbacks for p38 inhibitors are the isotype specificity and the undesired side effects often observed with kinase inhibitors. This study unveils a novel regulatory mechanism for p38α activation and signaling through arginine methylation and provides a new strategy, other than kinase inhibition, in intervening p38α signaling.

## 4. Materials and Methods

### 4.1. Materials and Plasmid

1-beta-D-arabinofuranosylcytosine (AraC) and benzidine were obtained from Sigma-Aldrich. S-adenosyl-L-[methyl-^3^H] methionine (^3^H-AdoMet, 0.55 mCi/ml, NET-155H) and fluorographic enhancer, EN^3^HANCE, were from PerkinElmer. The pFlag-CMV2-p38α plasmid [3] was used as a template for PCR to generate mutations on R49 and R149. These plasmids were subsequently cloned into the pET6H vector for expression of recombinant proteins.

### 4.2. Cell Culture

The human chronic myelogenous leukemia (CML) K562 cells were purchased from BCRC (Bioresource Collection and Research Center, Taiwan) and cultured in RPMI1640 medium supplemented with 10% fetal bovine serum, 100 IU/mL streptomycin and 100 IU/mL penicillin as described. The pLKO.1 puro-based shRNAs, including MKK3-sh1, MKK6-sh1, MKK6-sh2, MAPKAPK2-sh1, MAPKAPK2-sh2, were purchased from National RNAi Core Facility, Taiwan. The transfection of K562 was performed by using Lipofectamine^TM^ 2000 Reagent (Invitrogen). Stable clones of gene knockdown were selected in the presence of puromycin (0.5 μg/mL). The p38α-knockdown cell clones were generated by the same procedure as described [3,4]. The protein levels of p38α in these knockdown cells were around 20% of the parental cells.

### 4.3. Expression and Purification of Recombinant Proteins

Recombinant His-tagged p38α WT and mutants (R49K, R149K and R49/149K) proteins were expressed in *E. coli* BL21 (DE3) pLysS by isopropyl-beta-D-thiogalactoside (IPTG) (0.4 mM) induction and immobilized on Ni ^+^ -NTA agarose (Qiagen). Proteins were eluted with elution buffer (20 mM Tris-HCl pH7.9, 0.5 M NaCl, and 0.6 M imidazole) and dialyzed to remove imidazole. The purified proteins were stored in a buffer containing 20 mM Tris-HCl pH7.9 and 50 mM NaCl. The recombinant GST-fused PRMT1 proteins were expressed in *E. coli* BL21 by IPTG (0.2 mM) induction and immobilized on glutathione Sepharose 4B (GE Healthcare). Proteins were eluted with and stored in buffer (50 mM Tris-HCl Ph 7.9 and 20 mM reduced glutathione).

### 4.4. Immunoprecipitation

Cells were lysed in lysis buffer (150 mM NaCl, 20 mM Tris, pH7.4, 1% Triton X-100, 1 mM EDTA, 1 mM EGTA, 1 mM PMSF, 1 μg/mL leupeptin, 1 μg/mL aprotinin, 1 μg/mL pepstatin,1 mM Na_3_VO_4_, and 2.5 mM β-glycerophosphate). Cell lysates (1 mg) were incubated with 1 μL α-Flag antibodies (Sigma-Aldrich, F4042) in lysis buffer containing 0.2% Triton X-100 for 3 h at 4 °C followed by incubation with 20 μL protein G agarose-beads (GE Healthcare) for 1–3 hours at 4 °C. At the end of incubation, beads were washed four times with lysis buffer without Triton X-100. The phosphorylation states of p38α and the potential associated proteins were detected by Western Blotting. Alternatively, the immunoprecipitates were fractionated by SDS-PAGE and subjected to mass spectrometric analysis to examine the interactome.

To immunoprecipitate HA-PRMTs for methylation assay, cell lysates (0.5 mg) were incubated with α-HA antibodies (0.5 μL) (BioLegend, #901501) as described above and followed by incubation with 10 μL protein G agarose-beads (GE Healthcare). At the end of incubation, beads were washed twice with lysis buffer without Triton X-100, twice with PBS contained 0.2% tween-20, then twice with 25 mM Tris-HCl pH7.9. The immunoprecipitated HA-PRMTs were used immediately as an enzyme source for in vitro methylation assay.

### 4.5. In Vitro Methylation Assay

For the mass spectrometric identification of methylation sites in p38α, the methylation assay was carried out in vitro using recombinant wild-type His-p38α proteins as a substrate and with or without recombinant GST-PRMT1 proteins as an enzyme. The reactions were carried out using S-adenosyl-methionine (AdoMet, PerkinElmer) as a methyl donor as described previously [20]. Reactions were terminated and subjected to SDS-PAGE fractionation. The protein band containing p38α was excised from the gel and subjected to mass spectrometric analysis to examine its posttranslational modifications.

To compare methyl incorporation into the wild-type and mutant His-p38α proteins, HA-PRMT1, HA-PRMT1G80R, HA-PRMT6, HA-PRMT6KA proteins were expressed from the pcDNA3-HA2 plasmids in K562 cells and immunoprecipitated using anti-HA antibodies. Methylation reactions were carried out using S-adenosyl-L-[methyl-^3^H]methionine (^3^H-AdoMet, 1.65 μCi, PerkinElmer) as a methyl donor. Methyl incorporation was visualized by fluorography as described [20].

### 4.6. Mass Spectrometric Analysis

The gel pieces from SDS-PAGE were minced and incubated with trypsin (Promega). Peptides were extracted from the gel pieces by sonication in 50% acetonitrile containing 0.1% formic acid. The solutions were dried. The peptides were resuspended in 0.1% formic acid and analyzed by nanoflow high-performance liquid chromatography (Agilent Technologies 1200 series) followed by a LTQ-Orbitrap Discovery hybrid mass spectrometer with a nano-eletrospray ion source (Thermoelectron). This was performed by the Proteomics Center of National Yang- Ming University. The raw data were processed by the Xcalibur 2.0 SR1 software (Thermoelectron) and were converted to DTA files. Protein identity was determined by comparing the peptide sequences against the UniProtKB protein database (http://www.uniprot.org/) with TurboSequest search server T (version 27, revision 11) [36]. A protein was identified when two peptide ions matched with an Xcorr score >2.5. For the identification of p38α methylation sites, post-translational modifications (PTMs) of the peptides were identified by the in-house PTM finder program [37]. The peptides that contained PTM were matched with an Xcorr score >2.0. For the identification of associated proteins, quantitative analysis with MS spectra counting was performed by an in-house tool within a Microsoft VBA environment. MS spectra counts were normalized to total identified spectra per sample and then normalized to p38α spectra counts.

### 4.7. Limited Trypsin Digestion of Recombinant p38α

The recombinant His-tagged p38α WT and mutants (R49K, R149K and R49/149K) proteins (5 μg) were incubated with 0.5 μg trypsin (Sigma) in a final volume of 20 μL for 5, 15, and 30 min at 37 °C. Samples were analyzed by SDS-PAGE.

### 4.8. Erythroid Differentiation

For erythroid differentiation, K562 cells were treated with 1-beta-D-arabinofuranosylcytosine (AraC, 1μM, Sigma-Aldrich) for various times as indicated. Hemoglobin production was detected by using a benzidine/hydrogen peroxide solution as described [20]. Stained cells were counted under a light microscope. Two hundred cells were examined in each assay.

### 4.9. Real-Time Reverse Transcription PCR

Total RNA was extracted by using an illustra RNAspin Mini Isolation Kit (GE Healthcare). Isolated RNAs (4 μg) were subjected to cDNA synthesis with a RevertAid first strand cDNA synthesis kit (Fermentas). Real-time PCR was performed on an ABI StepOne Plus Real-Time PCR System (Applied Biosystems) using SYBR-Green reagents (SensiFAST SYBR Hi-ROX mix, Bioline). The samples were examined in triplicate for each assay and analyzed by using the comparative cycle threshold C_T_ (∆∆C_T_) method [3]. The expression of GAPDH (glyceraldehyde-3-phosphate dehydrogenase) was used as an internal control for cDNA contents. Gene-specific amplification was conducted with the following primer sets: for EKLF, 5’-CGGCAAGAGCTACACCAAG-3’ (sense) and 5’-CCGTGTGTTTCCGGTAGTG-3’ (antisense); for GATA1, 5’-CAGTCTTTCAGGTGTACCC -3’ (sense) and 5’-GAGTGATGAAGGCAGTGCAG-3’ (antisense); for ALAS2, 5’-GCAGCACTCAACAGCAAG-3’ (sense) and 5’-ACAGGACGGCGACAGAAA-3’ (antisense); for PBGD, 5’-CGCCTCCCTCTAGTCTCTGCTTCT-3’ (sense) and 5’- GTTGCCACCACACTGTCCGTCTG-3’ (antisense); for GAPDH, 5’-TGGTATCGTGGAAGGACTCATGAC-3’ (sense) and 5’-ATGCCAGTGAGCTTCCCGTTCAGC-3’ (antisense).

### 4.10. Western Blotting

Cells were lysed in RIPA buffer (150 mM NaCl, 10 mM Tris, pH7.4, 0.1% SDS, 1% Triton X-100, 5 mM EDTA, 1% Na-deoxycholate, 1 mM PMSF (phenylmethylsulfonyl fluoride), 1 μg/mL leupeptin, 1 μg/mL aprotinin, 1 μg/mL pepstatin). The primary antibodies used were α-Flag (Sigma-Aldrich, F4042, 1:2000 or Sigma-Aldrich, F7425, 1:1000), α-HA (BioLegend, #901501, 1:1000), α-p38 (Cell signaling, #9212, 1:1000), α-pp38 (Cell signaling, #9219, 1:1000), α-MKK3 (Cell signaling, #5674, 1:500), α-MKK6 (Abnova, #M02, 1:500), α-MAPKAPK2 (Cell signaling, #12155, 1:1000), α-PRMT1 (Sigma-Aldrich, #P16220, 1:1000), α-GAPDH (Cell signaling, G9545, 1:10000), α-mono- and di-methyl arginine (abcam, ab412, 1:500). The secondary antibodies used were anti-mouse (Sigma-Aldrich) or anti-rabbit (Genetex) IgG linked-horseradish peroxidase.

### 4.11. Statistical Analysis

All experiments were performed at least three times. The data are presented as means ± S.E.M. Statistical significance was performed by using Student’s t test, and *p* < 0.05 was considered statistically significant.

## Supplementary Materials

Supplementary materials can be found at https://www.mdpi.com/1422-0067/21/10/3546/s1.
