# Supplementary material for: The MKK-Dependent Phosphorylation of p38α Is Augmented by Arginine Methylation on Arg49/Arg149 during Erythroid Differentiation
Source: Int J Mol Sci. 2020 May 17;21(10):3546. doi: 10.3390/ijms21103546 (PMC7278938; doi:10.3390/ijms21103546)
Supplement: Supplementary file 1 [file ijms-21-03546-s001.pdf]

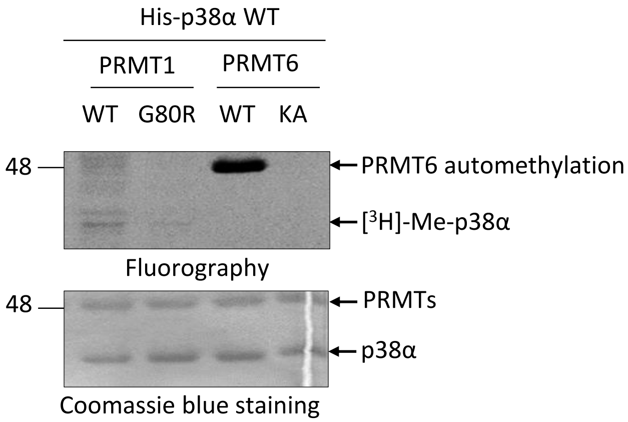

Figure S1. PRMT6 does not methylate p38α. HA-PRMT1, HA-PRMT1G80R, HA-PRMT6 and HA-PRMT6KA proteins were ectopically expressed in K562 cells and immunoprecipitated using anti-HA antibodies. The *in vitro* methylation assays were performed and methyl incorporation was visualized by fluorography as described in Methods. HA-PRMT1, but not HA-PRMT1G80R, methylated the recombinant p38α proteins. HA-PRMT6 was readily automethylated however did not methylate p38α. As expected, HA-PRMT6KA, a methyltransferase-defective mutant, did not methylated itself.

Figure S1

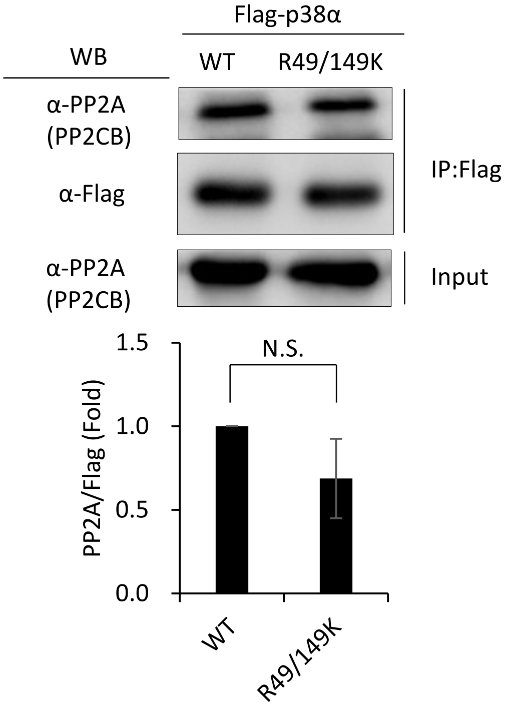

Figure S2. P38α is associated with protein phosphatase 2A and R49/149K mutation does not apparently affect the association. The Fla-p38α WT and R49/149K mutant proteins were immunoprecipitated using anti-Flag antibodies. Protein phosphatase 2A (PP2A) was associated with p38α and the R49/149K mutant to a comparable extent as examined by Western blotting.

Figure S2
